# *DDX60* Is Associated With Glioma Malignancy and Serves as a Potential Immunotherapy Biomarker

**DOI:** 10.3389/fonc.2021.665360

**Published:** 2021-06-10

**Authors:** Jingwen Zhang, Minjie Fu, Mengli Zhang, Jinsen Zhang, Zunguo Du, Hongyi Zhang, Wei Hua, Ying Mao

**Affiliations:** ^1^ Department of Neurosurgery, Huashan Hospital, Fudan University, Shanghai, China; ^2^ Department of Ultrasound, Hebei General Hospital, Shijiazhuang, China; ^3^ Department of Pathology, Huashan Hospital, Fudan University, Shanghai, China; ^4^ Department of Neurosurgery, Tangshan General Hospital, Tangshan, China; ^5^ Department of Neurosurgery, Tangshan Workers’ Hospital, Tangshan, China; ^6^ Neurosurgical Institute of Fudan University, Shanghai, China; ^7^ Shanghai Clinical Medical Center of Neurosurgery, Shanghai, China; ^8^ Neurosurgical Institute of Fudan University, Shanghai Key Laboratory of Brain Function Restoration and Neural Regeneration, Shanghai, China

**Keywords:** *DDX60*, glioma, biomarkers, immune checkpoints, *PD-L1*

## Abstract

*DDX60*, an interferon (IFN)-inducible gene, plays a promotional role in many tumors. However, its function in glioma remains unknown. In this study, bioinformatic analysis (TCGA, CGGA, Rembrandt) illustrated the upregulation and prognostic value of *DDX60* in gliomas. Immunohistochemical staining of clinical samples (n = 49) validated the DDX60 expression is higher in gliomas than in normal tissue (n = 20, *P* < 0.0001). It also could be included in nomogram as a parameter to predict the 3- and 5-year survival risk (C-index = 0.86). The biological process of *DDX60* in glioma was mainly enriched in the inflammatory and immune response by GSEA and GO analysis. *DDX60* expression had a positive association with most inflammatory-related functions, such as hematopoietic cell kinase (HCK) (R = 0.31), interferon (R = 0.72), STAT1 (R = 54), and a negative correlation with IgG (R = −0.24). Furthermore, *DDX60* expression tends to be positively related to multiple infiltrating immune cells, while negatively related to CD56 dim nature killer cell in glioma. Some important immune checkpoints, like *CTLA-4*, *PD-L1*, *EGF*, *CD96*, and *CD226*, were all positively related with *DDX60* (all Pearson correlation R > 0.26). The expression and correlation between DDX60, EGF, and PD-L1 were confirmed by western blot in clinical samples (n = 14, *P* < 0.0001) and GBM cells. These results indicated that *DDX60* might have important clinical significance in glioma and could serve as a potential immune therapeutic target.

## Introduction

Glioma is the most common malignancy in the brain, representing more than 70% of all central nervous system (CNS) malignancies (1). Glioblastoma multiforme (GBM), the most aggressive and malignant form of glioma, has a median survival of fewer than 21 months (2) despite the progress of neurosurgical resection, chemotherapy, radiation therapy, and novel approaches such as immunotherapy. Intratumoral heterogeneity widely exists in GBM (3) and has become an obstacle for molecular targeted therapy (4). To counteract the heterogeneity, therapies targeting the cytosolic innate immune receptors retinoic-acid inducible gene I (RIG-I) have been employed to gain a good response (5).

Acting as the upstream of RIG-I in the innate immune response, *DDX60* is a novel DEAD-box RNA helicase and first identified through microarray research of genes induced by measles’ virus infection in dendritic cells (DCs) (6). Through the helicase domain and ATP-binding site, *DDX60* can detect abnormal intracellular nucleic acids and then induce RIG-I-dependent type I interferons (type I IFNs) and other inflammatory cytokines (6–8). Besides, *DDX60* induced RIG-I-independent antiviral responses have also been demonstrated (8). Involved in RIG-I-dependent and independent innate immune responses, *DDX60* has been proven to be associated with the development of tumors (9–11). It was upregulated in oral squamous cell carcinoma and correlated with poor disease-free survival (10), while downregulated in colorectal cancer and related with the initiation and progression of the disease (11). Therefore, *DDX60* represents a potential target for tumor therapy. Immunotherapy and particularly immune checkpoint inhibitors, such as programmed death-ligand 1 (*PD-L1*) inhibitors, have revolutionized the treatment landscape of glioma (12). However, because of the heterogeneity and immunosuppression of glioma, some of the checkpoint inhibitor therapies fail to get a positive effect (1, 13), and new biomarkers for immune therapies are urgently needed. Herein, we assume that *DDX60* is a novel immune therapeutic target for glioma and explore its prognostic value and biological function in glioma.

This study demonstrated that *DDX60* is highly expressed in GBM and predicts poor prognosis of glioma by the Cancer Genome Atlas (TCGA), Chinese Glioma Genome Atlas (CGGA), Repository for Molecular Brain Neoplasia Data (REMBRANDT), and Gravendeel databases. Then, the correlation between *DDX60* expression and inflammatory responses, immune-related molecules, infiltrating immune cells as well as checkpoint protein in glioma was also established.

## Materials and Methods

### Data Collection

Glioma patient’s clinical information and gene expression data in the TCGA, CGGA, Rembrandt, and Gravendeel databases were downloaded from GlioVis (http://gliovis.bioinfo.cnio.es/) (14). The results shown here are in whole or part based upon data generated by the TCGA Research Network: https://www.cancer.gov/tcga. The expression information for *DDX60* in tumor and normal tissues in multiple cancers was acquired from UALCAN (https://ualcan.path.uab.edu/).

### Bioinformatics Analysis

The nomogram and calibration plots were constructed using the RMS package of R software. Pearson correlation and correlograms were generated using the circlize package and the corrgram package, respectively (15). Gene ontology (GO) analyses were employed to verify the biological processes by the R package of enrichplot and clusterProfiler (16). Gene set enrichment analysis (GSEA, http://software.broadinstitute.org/gsea/index) was performed between the *DDX60* high expression group and low expression group (17). The significant difference for GSEA was verified by the normalized enrichment score (NES) and false discovery rate (FDR). The related gene pathways with *P <*0.05 and FDR <0.1 were visualized by Cytoscape 3.7.2 version. The R package GSVA was used to search the enrichment status of inflammatory response-associated metagenes (18). Gene set associated with the immune function was extracted from the AmiGO 2 website (http://amigo.geneontology.org/amigo) to demonstrate the role of *DDX60* in the immune system in glioma. Genes with high correlation coefficients (R > 0.3 and *P* < 0.05) with *DDX60* were selected for heatmap displays. Metagenes of immune infiltration cells were downloaded from a previous study (19) and ssGSEA analysis was conducted *via* GSVA R package. Venn diagrams, boxplots, and heatmaps were generated using the Venn diagrams, ggplot2, and pheatmap packages in R software.

### Clinical Samples

Glioma tissues were gathered during 2019 to 2020 from patients (n = 60) who experienced craniotomy in the Department of Neurosurgery, Huashan Hospital of Fudan University. Normal brain tissues (n = 23) were obtained from traumatic brain injury patients who underwent partial resection as decompression treatment. These experimental protocols were approved by the Human Ethics Committee of Huashan Hospital and informed consent was collected from all patients.

### Knockdown

SiRNA targeted human DDX60 (siRNA#1, CCAUCUGCCUCUUUCUCAATT; and siRNA#2, GGAUUUGAUGAGUUGGCAATT) and control siRNA were obtained from Hanbio (Shanghai, China). SiRNA knockdown of DDX60 was performed with Invitrogen Lipofectamine 2000 and standard procedures (20).

### Immunohistochemical Staining

IHC staining was performed as described previously (10). The sections were incubated with rabbit anti-human *DDX60* Ab (1:200, Abcam) as the primary antibody. Immune reactive score (IRS) was conducted as described (21).

### Western Blot

The glioma and normal brain tissues were minced by scissors and homogenized in RIPA lysis buffer with proteinase inhibitors, and the homogenate was centrifuged at 13,000 g, 4°C for 10 min, and the supernatant was collected. Cell protein was extracted using RIPA lysis buffer for 20 min at 4°C. Then the 5× loading buffer was added, and the sample was boiled for 5 min. Western blot was performed as previously described (11). The primary antibodies include rabbit anti-human *PD-L1* Ab (1:1,000, Abcam), anti-*DDX60* Ab (1:1,000, Abcam), and anti-GAPDH (1:50,000, Proteintech). The data analysis as well as statistics was performed through ImageJ as described previously (22).

### Statistical Analysis

R language 3.6.2 version was employed to perform statistical analysis. A Student’s t-test was conducted to evaluate *DDX60* expression differences. ‘Survival’ and ‘survminer’ packages in R were used for survival analysis. Continuous variables of the *DDX60* expression were dichotomized by conducting the best cutoff values detected by the “surv_cutpoint” function of the “survminer” R package (23). the statistical significance was calculated by the log-rank test (24). Univariate and multivariate Cox proportional hazards models were performed to search hazard ratios (HRs) by R.

## Results

### The Overexpression of *DDX60* Is Correlated With Malignancy in Gliomas

**Figure 1:**
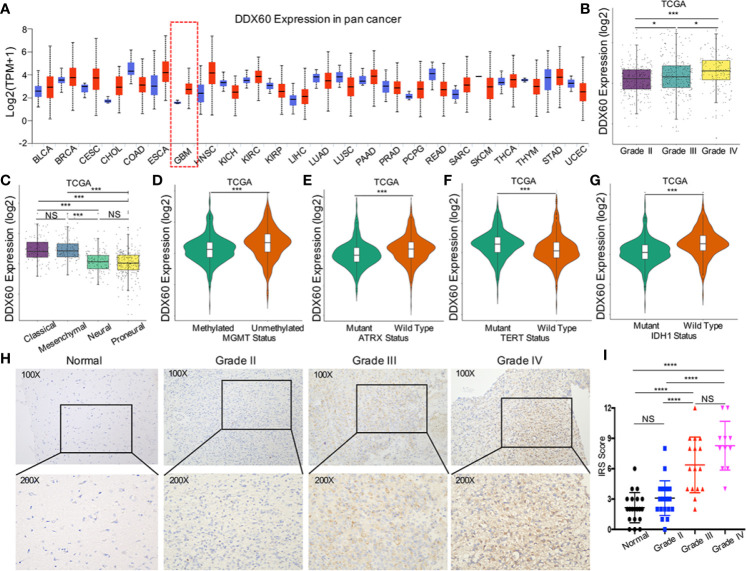
*DDX60* expression upregulated in glioma and was correlated with glioma biomarkers. **(A)** The expression information for *DDX60* in tumor and normal tissues in multiple cancers in UALCAN. Blue represents the normal tissue and red represents the tumor tissue. **(B)** *DDX60* expression level increase along with WHO grade in the TCGA database. **(C)** Comparison between different subtypes of GBM in TCGA. **(D–F)** *DDX60* was upregulated in ATRX wild-type group, MGMT unmethylated group and TERT expressed group based on TCGA dataset. **(G)** *DDX60* was significantly overexpressed in IDH wild-type glioma based on TCGA dataset. **(H)** Representative IHC staining of *DDX60* in normal brain tissue and WHO grade II–IV gliomas. **(I)** Immune reactive score (IRS) of *DDX60* in normal brain tissue and different WHO grade gliomas, Normal (n = 20), Grade II (n = 22), Grade III (n = 16), Grade IV (n = 11). *P < 0.05, ***P < 0.001 and ****P < 0.0001. NS, not significant.

The expression of *DDX60* was upregulated in multiple cancers comparing with normal tissues, including GBM (**Figure 1A**). TCGA and Rembrandt dataset analysis showed that *DDX60* is overexpressed in glioma (*P* < 0.001, **Supplementary Figures 1A, B**). As expected, TCGA analysis demonstrated that *DDX60* in GBM is higher than in lower-grade glioma (LGG, grades II and III, *P* < 0.0001 and *P* = 0.032, respectively, **Figure 1B**). The CGGA and Rembrandt dataset also confirmed the lower levels of *DDX60* in LGG (**Supplementary Figures 1C, D**). Additionally, *DDX60* favored its expression in mesenchymal and classical subtype (**Figure 1C** and **Supplementary Figure 1E**), *MGMT* unmethylated (*P* < 0.001, **Figure 1D**), *ATRX* wild-type (*P* < 0.001, **Figure 1E**), *TERT* promoter mutated (*P* < 0.001, **Figure 1F**), and *IDH* wild-type gliomas (*P* < 0.001, **Figure 1G**, **Supplementary Figure 1F**). All the *in silico* analyses demonstrated the correlation between *DDX60* and the malignancy of gliomas.

IHC analysis (**Figures 1H, I**) verified that *DDX60* expression in normal tissue (mean IRS = 2.15, n = 20) was lower than in glioma tissue (mean IRS = 5.33, n = 49) (*P* < 0.0001). *DDX60* was predominantly expressed in the cytoplasm of glioma cells and expressed distinctively in different WHO grades. The expression of *DDX60* in grade II (mean IRS = 3.09, n = 22) was significantly lower than that in grade III (mean IRS = 6.38, n = 16, *P* < 0.0001) and in grade IV (mean IRS = 8.27, n = 11, *P* < 0.0001), while no statistical difference was found between grade III and grade IV (*P* = 0.077). In summary, *DDX60* expression was higher in glioma than in normal tissue and increased with malignant escalation of glioma.

### *DDX60* Could Predict a Poor Prognosis of Gliomas

**Figure 2:**
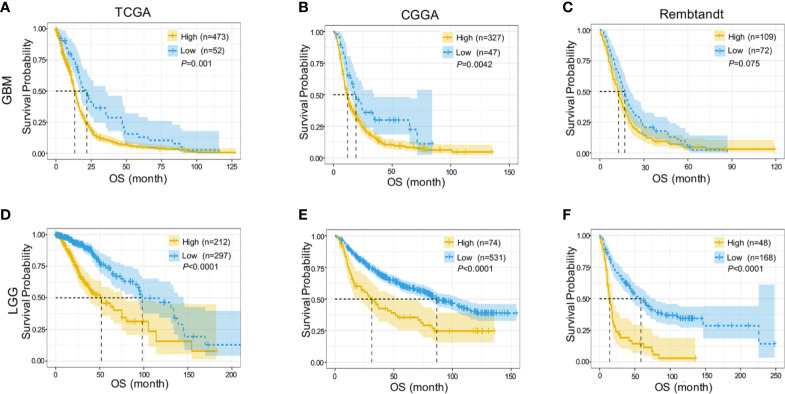
*DDX60* predicts poor prognosis of glioma patients. **(A–C)** Higher *DDX60* expression portended poor prognosis for GBM in TCGA database, CGGA dataset and Rembrandt dataset. **(D–F)** LGG patients with increased expression of *DDX60* also get poor prognosis in TCGA database, CGGA dataset and Rembrandt dataset.

We further assessed the prognostic value of *DDX60* in both LGG and GBM based on TCGA, CGGA, and Rembrandt datasets. Higher *DDX60* expression seemed to portend a poor prognosis for GBM in TCGA (*P* = 0.001, **Figure 2A**). Likewise, a strong correlation between higher expression of *DDX60* and worse OS for GBM patients was detected in CGGA and Rembrandt datasets, respectively (*P* = 0.0042 and *P* = 0.075, **Figures 2B, C**). Survival data in LGG were consistent with those in GBM in TCGA, CGGA, and Rembrandt, respectively (all *P* < 0.0001, **Figures 2D–F**). These outcomes demonstrated *DDX60* as a negative prognostic indicator in gliomas.

**Figure 3:**
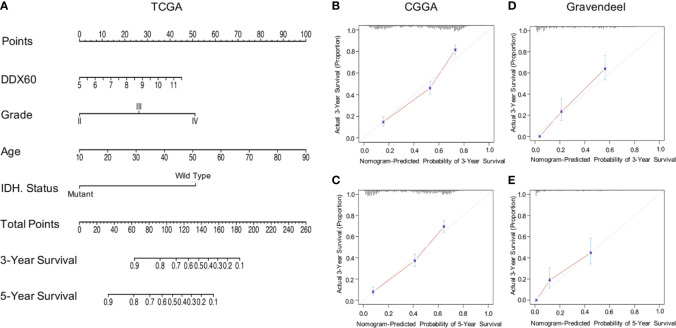
*DDX60*-related prediction nomogram. **(A)** Nomogram for predicting 3- or 5-year survival in glioma patients. The top row represents the point value for each variable. Rows 2–5 display the variables included in the nomogram. Each variable fits to a point value based on glioma characteristics. The Total Points axis equals to the sum of the point values, and the lines downward to the total points is used to establish the liability of 3- or 5-year survival. **(B, C)** Calibration curves for predicting patient survival in CGGA dataset at 3 and 5 years. **(D, E)** The Gravendeel Dataset was also used as the validation cohort to show calibration curves for predicting patient survival at 3 and 5 years.

Univariate (HR = 1.623, *P* < 0.0001) and multivariate (HR = 1.1937, *P* = 0.024) Cox regression analyses were then performed, and factors related to the prognosis of gliomas were selected (**Table 1**). The prognostic nomogram with a risk classification system for 3- and 5-year survival rates of glioma based on TCGA was established (n = 596, **Figure 3A**). This nomogram integrated all significant independent variables including *DDX60*, and the C-index for OS prediction was 0.86. The calibration plot for the probability of survival at 3 or 5 years based on the two independent cohorts of CGGA (n = 960) and Gravendeel (n = 216) showed optimal conformity between the prediction by nomogram and actual observation (**Figures 3B–E**). The demographics and clinical characteristics of patients with glioma in primary and validation cohort were in **Supplementary Table 1**.

**Table 1:** Univariate and multivariate analysis of overall survival in the TCGA database.

Variables	Multivariate				Univariate			
	HR	Lower	Upper	*P*	HR	Lower	Upper	*P*
		0.95	0.95			0.95	0.95	
*DDX60*	1.1937	1.0236	1.392	0.024	1.623	1.417	1.859	<0.0001
Gender								
Female	Reference				Reference			
Male	1.0008	0.7543	1.328	0.99549	1.169	0.8911	1.533	0.26
Age	1.0324	1.0199	1.0451	<0.0001	1.069	1.058	1.08	<0.0001
WHO Grade								
Grade II	Reference				Reference			
Grade III	1.9525	1.2418	3.07	0.00376	2.898	1.9	4.419	<0.0001
Grade IV	3.6728	2.1263	6.3442	<0.0001	18.232	11.99	27.721	<0.0001
IDH Status								
Wild-type	Reference				Reference			
Mutation	0.2704	0.1758	0.4158	<0.0001	0.09983	0.07469	0.1334	<0.0001

### *DDX60* Seems to Contribute to Multiple Biological Processes in Gliomas

**Figure 4:**
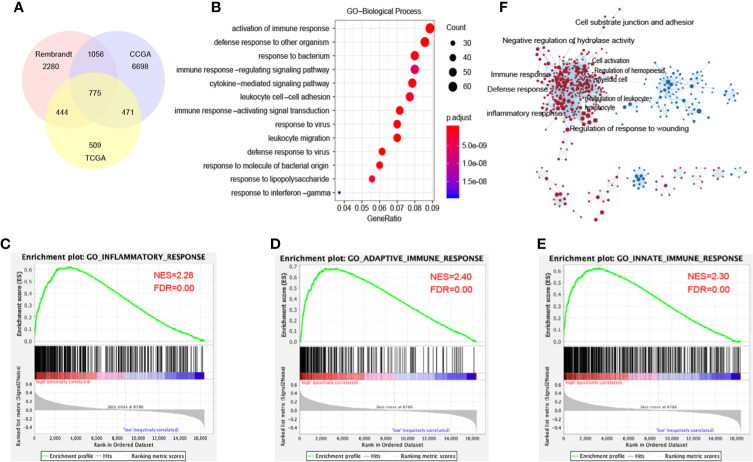
*DDX60*-related biological processes in glioma. **(A)** 775 related genes of *DDX60* were chosen in glioma from the TCGA, CGGA, and Rembrandt databases based on Pearson’s correlation analysis (|R| > 0.3). **(B)** The term of immune response enriched most through gene ontology (GO) analysis on biological processes (BP). **(C–E)** GSEA employed to verify the gene signatures, mainly included inflammatory response, adoptive immune response, and innate immune response. **(F)** The cytoscape of enrichment map results. Nodes represent gene-sets, which were automatically arranged so that highly similar gene-sets are placed close together, and node size represents the number of genes in the gene-set.

*DDX60* is known as an IFN-inducible gene (6). To verify the function of *DDX60* in gliomas, 775 genes were identified in the intersection of the three datasets through Pearson’s correlation (|R| > 0.3, **Figure 4A**, **Supplementary Table 2**). Gene ontology (GO) analysis illustrated that *DDX60* was involved in multiple biological processes, including immune response, defense response to other organisms, cytokine-mediated signaling pathway (**Figure 4B**). Meanwhile, GSEA verified the gene signatures were mainly enriched in the inflammatory response and immune response (**Figures 4C–E**). The Cytoscape of enrichment map displayed that enriched terms are centrally attached to the immune response as well as inflammatory response (**Figure 4F**).

### *DDX60* Is Highly Related to Inflammatory Responses and Immune Functions

**Figure 5:**
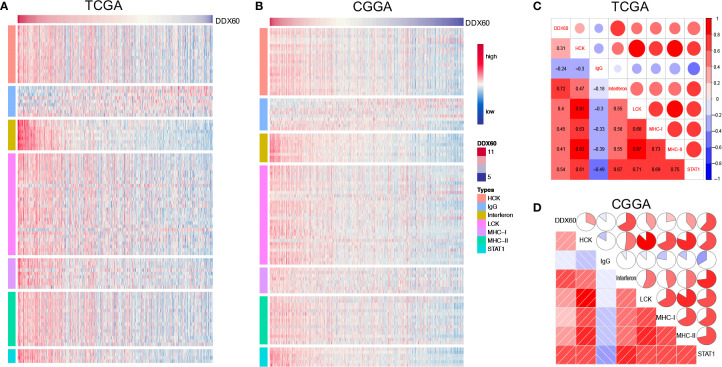
*DDX60*-related inflammatory responses in glioma. **(A, B)** Heatmap of the correlation between *DDX60* and metagenes based on TCGA and CGGA. **(C, D)** Correlogram showed the association between *DDX60* and seven inflammatory-related metagenes in TCGA and CGGA datasets.

To better understand *DDX60*-related inflammatory responses in glioma, seven metagenes including 105 genes (**Supplementary Table 3**) associated with multiple types of inflammation and immune functions were chosen (25). Clustering based on TCGA and CGGA showed that all clusters have a positive correlation with *DDX60* expression level apart from IgG (**Figures 5A, B**). Correlograms show that *DDX60* expression level had a positive association with hematopoietic cell kinase (HCK), interferon, lymphocyte-specific protein tyrosine kinase (LCK), major histocompatibility complex class-I (MHC-I), major histocompatibility complex class-II (MHC-II), and STAT1, while it had a negative correlation with IgG (**Figures 5C, D**). Among the gene set associated with the immune function, 103 out of 105 genes in TCGA and 145 out of 149 genes in CGGA were significantly positively associated with *DDX60* (**Supplementary Figure 2**, **Supplementary Tables 4** and **5**). Collectively, a strong association between *DDX60* expression patterns and immune functions has been found in glioma.

**Figure 6:**
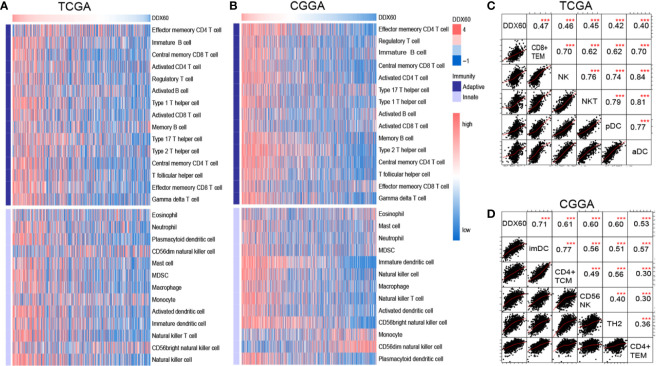
*DDX60* is closely correlated with immune cells in the glioma microenvironment. **(A, B)** Heatmap based on TCGA and CGGA dataset visualizing the relationship between *DDX60* and 28 infiltrating immune cell populations. **(C, D)** Correlation matrixes of the top five most related immune cells with *DDX60* in TCGA and CGGA datasets. ***P < 0.001.

Metagenes (**Supplementary Table 6**) (19) were delineated in heatmaps to show the correlation between *DDX60* expression and 28 infiltrating immune cell populations (**Figures 6A, B** and **Supplementary Figure 3A**). The top five *DDX60*-related immune cells in TCGA were effector memory CD8 T cell (CD8+ TEM), natural killer cell (NK), natural killer T cell (NKT), plasmacytoid dendritic cell (pDC), and activated dendritic cell (aDC) (**Figure 6C**). Correlation matrixes of the top five most related immune cells in the CGGA and Rembrandt dataset were also displayed (**Figure 6D** and **Supplementary Figure 3B**), and all the Pearson’s correlation coefficient (R) and P-values were listed (**Supplementary Table 7**). In summary, *DDX60* expression tends to be positively related to most infiltrating immune cells, while negatively related with CD56 dim nature killer cell in glioma.

### *DDX60* Is Strongly Correlated With Immune Checkpoint Proteins

**Figure 7:**
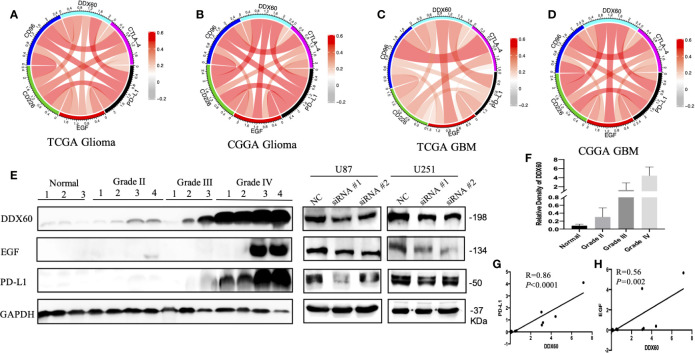
Relationship between *DDX60* and checkpoint markers in glioma. **(A–D)** The associations between *DDX60* and immune check point-related genes including *CTLA-4*, PD-L1, *EGF*, *CD96*, and *CD226* based on TCGA and CGGA datasets were presented. **(E)** The level of DDX60, PD-L1 and EGF protein expression in normal brain tissue, WHO grade II–IV glioma tissue and DDX60 knockdown glioma cells were shown by western blot. **(F)** The relative density of DDX60 of western blot. **(G)** A strong association between DDX60 and PD-L1 (R = 0.86, *P* < 0.0001) according to the gray-scale analysis of the western blot. **(H)** The expression correlation between EGF and DDX60 (R = 0.56, *P* = 0.002).

The correlation between *DDX60* and some important checkpoint proteins like *CTLA-4*, *PD-L1*, *EGF*, *CD226*, and *CD96* was assessed in the TCGA and CGGA datasets. Circos plots showed the strong positive association between *DDX60* and these five immune check point-related genes in all grade gliomas (**Figures 7A–D**). The correlation coefficients (R) between *DDX60* and immune checkpoint genes were shown in **Table 2**. Among these genes, *PD-L1* showed the strongest positive correlation with *DDX60* in the TCGA dataset (glioma R = 0.54, GBM R = 0.45). Western blot analysis with clinical samples and DDX60 knockdown glioma cells demonstrated that *DDX60* protein expression was correlated with *PD-L1* (*P* < 0.0001, R = 0.86) and *EGF* (*P* = 0.002, R = 0.56) (**Figures 7E–H**). The relatively density of PD-L1 and EGF of western blot for glioma tissue were shown in supplementary **Figure 4**. Results predicted the possible synergistic effects of *DDX60* with these checkpoint genes.

**Table 2:** The R values between *DDX60* and immune checkpoint genes.

Immune checkpoint gene	TCGA-Glioma	TCGA-GBM	CGGA-Glioma	CGGA-GBM
*PD-L1*	0.54	0.45	0.61	0.58
*CTLA-4*	0.33	0.26	0.30	0.35
*EGF*	0.38	0.47	0.52	0.55
*CD226*	0.45	0.33	0.62	0.60
*CD96*	0.46	0.42	0.44	0.43

## Discussion

Although novel therapies such as immunotherapy have been used, less progress has been made in overall survival (OS) in GBM patients (26). Within-tumor heterogeneity is a major driver of progression, recurrence, and therapeutic resistance of GBM (27). Therefore, more biomarkers are needed to be identified to accurately access the prognosis of GBM patients and individualize treatment strategies.

*DDX60* is a DEAD-box RNA helicase and has been proved upregulated in melanoma (28) and oral squamous cell carcinoma (OSCC) (10) while deregulating in colorectal cancer (11). Herein, we demonstrated that higher expression of *DDX60* was correlated with high-grade glioma. Moreover, we observed that *DDX60* was significantly overexpressed in glioma with *IDH* wild-type, *ATRX* wild-type, *MGMT* unmethylated as well as *TERT* promoter mutated. These findings suggest that gliomas with high *DDX60* expression have increased malignancy and insensitive to chemotherapy (29).

A previous study has shown that high expression of *DDX60* was significantly associated with poor survival in lip squamous cell carcinoma (10). As expected, this study demonstrated that high *DDX60* expression was associated with poor prognosis both in LGG and GBM. As the nomogram could show better performance than conventional staging systems and more precise prognostic forecast in some tumors (30, 31), we identified *DDX60* as a prognostic marker of glioma and built a nomogram with a risk classification system. The four parameters included in the nomogram complied with clinical relevance and Cox analysis (32). Studies have shown that gender, age, WHO grade, and IDH status are related to the prognosis of glioma (33–35). However, our univariate and multivariate Cox analyses did not find a correlation between sex and prognosis of glioma. Thus, gender was excluded from the visualization of the nomogram. The calibration plots of the two external validation cohorts were highly fitted, illustrating that the nomogram performed well in predicting 3- or 5-year survival for glioma patients.

GO and GSEA of *DDX60* in this research showed that immune and inflammatory responses were the most enriched terms. It has been indicated that inflammation regulates various stages of the tumor process, such as promotion and invasion (36). Different proinflammatory mediators induced by inflammation promote tumor progression by regulating chemokines, cascades of cytokines, adhesion, and pro-angiogenic activities (37). *DDX60* is an IFN-inducible gene, and its ectopic expression can promote RIG-I RNA-binding activity, causing RIG-I-mediated type I IFN expression (6). Type I IFNs (IFN-alpha and IFN-beta) are a family of cytokines with a diverse cellular processes such as regulation of inflammatory and immune responses (38). Through mediating type I IFNs, *DDX60* can also activate STAT1and upregulate MHC-I (39); these results are compatible with our findings. Furthermore, we also found that *DDX60* was positively associated with LCK and HCK while negatively associated with IgG response; these results refined the mechanism of *DDX60* in the inflammatory response of glioma.

Multiple non-neoplastic cells exist in the GBM microenvironment, such as infiltrating immune cells (40). The immune surveillance of these immune cells would be converted to detrimental function when the immune system is overwhelmed by cancer burden during tumor development (40). The high correlation between infiltrating immune cells and *DDX60* expression has been demonstrated in this study, such as type 17 T helper cell (Th17) and macrophages. Previous research had verified that Th17 cells in the GBM microenvironment may participate in immune suppression *via* TGF-*β*1-induced IL-10 secretion (41). Tumor-associated macrophages (TAMs) in GBM have been proven to be the dominant infiltrating immune cell population and engaged in interactions with tumor cells to aid tumor infiltration and proliferation (42). These results suggest that *DDX60* might involve in immunosuppression by mediating immune cells in glioma.

CNS used to be considered as “immunologically privileged” in the past decades. However, as more and more researchers have verified that the leukocyte lymphatics not only be present in CNS but also have the ability to transport antigens to cervical lymph nodes (43–46), researchers agree that CNS is more likely “immunologically unique” rather than “immunologically privileged”. These provide a basis for glioma immunotherapy. Undoubtedly, immunotherapy holds a bright future for the treatment of glioma. However, it seems difficult to achieve stable and better outcomes for immunotherapy in clinical. This can be mostly attributed to the tumor heterogeneity of glioma (47, 48). New biomarkers which can predict and monitor immunotherapy response have become urgently needed. In this study, *DDX60* was not only a prognosis prediction for glioma patients but also an indicator of the immune microenvironment of glioma and might become a novel biomarker and potential therapeutic target.

Checkpoint inhibitors, advancing rapidly in recent years, have been the immunotherapy most advanced in clinical use. Among them, death protein 1 (PD-1) and PD-L1 are the most broadly studied (49). PD-L1 is widely expressed on the GBM infiltrating T cells and is a negative prognosticator for GBM outcome (50). PD-1 combining with PD-L1, negatively modulates T cell receptor-induced signaling transduction, blocks the activation of cytotoxic T cell, and inhibits the producing of inflammatory factors, causing T cell inability (49). Many clinical trials in GBM are evaluating anti-PD-L1 agents such as Durvalumab, Atezolizumab, Avelumab, alone or combined with other therapies (51). However, not all the research studies on anti-PD-L1 agents come out with a meaningful benefit (51). Thus, new biomarkers that can accurately predict the efficacy of *PD-L1* inhibitor therapy are urgently needed. In this study, we demonstrated that the correlation coefficients (R) between *PD-L1* and *DDX60* were 0.54 in the TCGA glioma dataset and 0.61 in the CGGA glioma dataset. We further performed western blot both in patient tissues and glioma cell lines to verify the strong correlation between PD-L1 and DDX60 (*P* < 0.0001, R = 0.86). The mechanism of the positive correlation between DDX60 and PD-L1 might lie in the IFN/PD-L1 axis. As an IFN-inducible gene, the ectopic expression of DDX60 can improve RIG-I RNA-binding activity, causing RIG-I-mediated IFN expression (6). A previous study has shown that IFN was a crucial factor of PD-L1 expression in the glioma model (52). Thus, the upregulation of DDX60 might lead to a higher expression level of PD-L1. These results implied that glioma patients with higher *DDX60* expression might benefit more from PD-L1 blocker therapy.

Besides, the correlation between *DDX60* and some other immune checkpoint genes such as cytotoxic T-lymphocyte-associated antigen-4 (CTLA-4), epidermal growth factor (EGF), CD226, and CD96 was shown (**Figure 7**, **Table 2**). As the correlation coefficients (R) between *EGF* and *DDX60* were >0.5 in both CGGA glioma and CGGA GBM database, western blot was then employed to demonstrate the strong association between EGF and DDX60 (*P* = 0.002, R = 0.56). Thus, these results illustrate the predictive significance and potential synergistic responses of *DDX60* to immune checkpoint treatments.

There have been reports that *DDX60* was overexpressed in other types of cancers (10, 11), but most of them did not further explore the intrinsic mechanisms. Thus, the novelties of this paper lie not merely in the findings of prognostically significant of *DDX60* in glioma, but also in the mechanism of *DDX60* on glioma. Our research verified the strong association between *DDX60* and glioma immune microenvironment, clarified the mechanism of *DDX60*, and proposed that *DDX60* might become a novel biomarker for immunotherapy.

In conclusion, these results would widen our knowledge of the expression and prognostic value of *DDX60* in gliomas. Furthermore, as a potential therapeutic target, *DDX60* is positively correlated with *PD-L1* and other checkpoints. Thus, these findings will help to optimize the immunotherapy in glioma.

## Data Availability Statement

Publicly available datasets were analyzed in this study. This data can be found here: http://gliovis.bioinfo.cnio.es/.

## Ethics Statement

The studies involving human participants were reviewed and approved by the Human Ethics Committee of Huashan Hospital. The patients/participants provided their written informed consent to participate in this study.

## Author Contributions

JingZ designed and conducted the study and drafted the original manuscript. MF, JinZ, and MZ helped collect the databases. ZD helped conduct the IHC and IRS. WH, HZ, and YM supervised the study. WH revised the manuscript. All authors contributed to the article and approved the submitted version.

## Funding

The study was funded by Shanghai Science and Technology Commission (17430750200), the National Natural Science Foundation of China (82072785, 82072784), Join Breakthrough Project for New Frontier Technologies of Shanghai Hospital Development Center (SHDC12016120).

## Conflict of Interest

The authors declare that the research was conducted in the absence of any commercial or financial relationships that could be construed as a potential conflict of interest.

## Abbreviations

GBM, glioblastoma multiforme; OS, overall survival; MHC I, major histocompatibility complex class I; GO, Gene ontology; BP, biological processes; PD-1, programmed cell death protein 1; *PD-L1*, programmed death-ligand 1; *CTLA-4*, cytotoxic T-lymphocyte-associated antigen-4; Rembrandt, Repository for Molecular Brain Neoplasia Data; TCGA, The Cancer Genome Atlas; HRs, hazard ratios.
