# Supplementary material for: DDX60 Is Associated With Glioma Malignancy and Serves as a Potential Immunotherapy Biomarker
Source: Front Oncol. 2021 Jun 10;11:665360. doi: 10.3389/fonc.2021.665360 (PMC8222729; doi:10.3389/fonc.2021.665360)
Supplement: Supplementary file 1 [file DataSheet_1.zip › supplement/Supplementary Figures.docx]

Supplementary Figure 1. (A, B) The expression level of DDX60 is upregulated in both GBM samples and glioma samples. (C, D) DDX60 expression level increase along with WHO grade in the CGGA and Rembrandt databases. (E) Comparison between different subtypes of GBM in Rembrandt database. (F) DDX60 was significantly overexpressed in IDH wildtype glioma based on TCGA, CGGA and Gravendeel datasets.

**Supplementary Figure 2. Heatmap of the correlation between DDX60 and immune function-related genes in glioma.** The relationship between DDX60 and glioma IDH status, WHO grade, and immune response-related genes are presented. Gene data were downloaded from the AmiGO 2 website for TCGA (**A**) and CGGA (**B**) databases.

**Supplementary Figure 3.** DDX60 is closely correlated with immune cells in the glioma microenvironment**. (A)** Heatmap based on Rembrandt dataset visualizing the relationship between DDX60 and 28 infiltrating immune cell populations. **(B)** Correlation matrixes of the top five most related immune cells with DDX60 in Rembrandt dataset.

**Supplementary Figure 4.** The relatively density of PD-L1 and EGF of western blot for glioma tissue（Related to Figure 7）.

**Supplementary table 1. Demographics and clinical characteristics of patients with glioma**

| Characteristic | Primary cohort | Validation cohort | |
| --- | --- | --- | --- |
|  | TCGA(n=596) | CGGA(n=960) | Gravendeel (n=216) |
| DDX60 level |  |  |  |
| Median | 8.389 | 1.6758 | 7.837 |
| Range | 5.299-11.408 | -1-4.9255 | 4.813 -10.176 |
| WHO grade |  |  |  |
| Grade II | 211 | 274 | 22 |
| Grade III | 239 | 308 | 66 |
| Grade IV | 146 | 378 | 128 |
| Age |  |  |  |
| Median | 47 | 43 | 51 |
| Range | 14-89 | 8-79 | 14-81 |
| IDH1.status |  |  |  |
| Wild type | 220 | 431 | 136 |
| Mutant | 376 | 529 | 80 |

**Supplementary Table 7. The Pearson’s correlation coefficient (*r*) and *p*-values between DDX60 expression and immune cells**

TCGA Dataset

| Cell | R | *P* value |
| --- | --- | --- |
| Effector memory CD8 T cell | 0.4742873 | 8.00E-39 |
| Natural killer cell | 0.46329206 | 6.71E-37 |
| Natural killer T cell | 0.45102202 | 7.81E-35 |
| Plasmacytoid dendritic cell | 0.42408621 | 1.39E-30 |
| Activated dendritic cell | 0.40219857 | 2.11E-27 |
| MDSC | 0.39304278 | 3.87E-26 |
| Type 1 T helper cell | 0.39222535 | 4.99E-26 |
| Type 17 T helper cell | 0.39078561 | 7.80E-26 |
| Immature B cell | 0.38551647 | 3.94E-25 |
| T follicular helper cell | 0.383981 | 6.28E-25 |
| Macrophage | 0.38034109 | 1.88E-24 |
| Immature dendritic cell | 0.36807662 | 6.83E-23 |
| Central memory CD4 T cell | 0.36623667 | 1.15E-22 |
| Central memory CD8 T cell | 0.36037727 | 6.02E-22 |
| Gamma delta T cell | 0.33993802 | 1.47E-19 |
| Activated CD8 T cell | 0.3161763 | 5.36E-17 |
| Activated B cell | 0.31006418 | 2.25E-16 |
| Mast cell | 0.2887856 | 2.57E-14 |
| Neutrophil | 0.28362491 | 7.64E-14 |
| Regulatory T cell | 0.27604515 | 3.64E-13 |
| Activated CD4 T cell | 0.24703684 | 9.27E-11 |
| Type 2 T helper cell | 0.18223227 | 2.09E-06 |
| Effector memory CD4 T cell | 0.18119591 | 2.39E-06 |
| Memory B cell | 0.11059592 | 0.00418317 |
| CD56bright natural killer cell | 0.08809741 | 0.02267815 |
| Eosinophil | 0.08264686 | 0.03257166 |
| Monocyte | 0.06541623 | 0.09090555 |
| CD56dim natural killer cell | -0.0642925 | 0.09660567 |

CGGA dataset

| Cell | R | *P* value |
| --- | --- | --- |
| Immature dendritic cell | 0.71426718 | 1.21E-159 |
| Central memory CD4 T cell | 0.61430589 | 1.20E-106 |
| CD56bright natural killer cell | 0.59727452 | 1.92E-99 |
| Type 2 T helper cell | 0.59522681 | 1.32E-98 |
| Effector memory CD4 T cell | 0.53143295 | 2.84E-75 |
| Natural killer T cell | 0.51121713 | 7.20E-69 |
| Activated CD4 T cell | 0.50869908 | 4.22E-68 |
| Gamma delta T cell | 0.50755767 | 9.36E-68 |
| Plasmacytoid dendritic cell | 0.49083519 | 7.81E-63 |
| T follicular helper cell | 0.49018485 | 1.20E-62 |
| Central memory CD8 T cell | 0.4659742 | 5.28E-56 |
| Natural killer cell | 0.46481805 | 1.06E-55 |
| CD56dim natural killer cell | -0.4217384 | 3.66E-45 |
| Memory B cell | 0.40318205 | 4.49E-41 |
| Type 1 T helper cell | 0.3934333 | 5.00E-39 |
| Activated dendritic cell | 0.37482207 | 2.64E-35 |
| Regulatory T cell | 0.37042408 | 1.84E-34 |
| Immature B cell | 0.35835453 | 3.29E-32 |
| Macrophage | 0.3581271 | 3.63E-32 |
| MDSC | 0.27854567 | 1.35E-19 |
| Mast cell | 0.2645281 | 9.24E-18 |
| Activated CD8 T cell | 0.23160249 | 7.30E-14 |
| Activated B cell | 0.20411938 | 4.90E-11 |
| Effector memory CD8 T cell | 0.17413273 | 2.25E-08 |
| Neutrophil | 0.168088 | 6.86E-08 |
| Type 17 T helper cell | 0.16322213 | 1.64E-07 |
| Monocyte | -0.063138 | 0.04400703 |
| Eosinophil | 0.06075929 | 0.05262304 |

Rembrandt dataset

| Cell | R | *P* value |
| --- | --- | --- |
| CD56bright natural killer cell | 0.52410643 | 3.10E-42 |
| Central memory CD4 T cell | 0.51708643 | 5.69E-41 |
| Immature dendritic cell | 0.49063531 | 1.83E-36 |
| Central memory CD8 T cell | 0.48040804 | 8.01E-35 |
| Activated dendritic cell | 0.47673665 | 3.01E-34 |
| Gamma delta T cell | 0.47181557 | 1.73E-33 |
| Effector memory CD8 T cell | 0.47117227 | 2.18E-33 |
| Activated CD8 T cell | 0.46834762 | 5.86E-33 |
| Plasmacytoid dendritic cell | 0.46467468 | 2.09E-32 |
| MDSC | 0.46294874 | 3.79E-32 |
| Macrophage | 0.45757695 | 2.35E-31 |
| Natural killer cell | 0.45522413 | 5.17E-31 |
| Natural killer T cell | 0.43613553 | 2.49E-28 |
| Regulatory T cell | 0.41221509 | 3.37E-25 |
| Type 1 T helper cell | 0.40270169 | 5.06E-24 |
| Mast cell | 0.3644462 | 1.17E-19 |
| Activated CD4 T cell | 0.35735225 | 6.53E-19 |
| Immature B cell | 0.27315458 | 2.20E-11 |
| Memory B cell | 0.16369307 | 7.48E-05 |
| Neutrophil | -0.1473446 | 0.0003704 |
| Effector memory CD4 T cell | 0.14159933 | 0.00062609 |
| T follicular helper cell | 0.12290804 | 0.00302801 |
| Eosinophil | 0.09144675 | 0.0276516 |
| Activated B cell | 0.06017517 | 0.14778618 |
| Type 17 T helper cell | 0.03600447 | 0.38675753 |
| CD56dim natural killer cell | -0.028701 | 0.49028011 |
| Type 2 T helper cell | 0.02862394 | 0.49144671 |
| Monocyte | 0.02836566 | 0.49536612 |
